# RNA secondary structure profiling in zebrafish reveals unique regulatory features

**DOI:** 10.1186/s12864-018-4497-0

**Published:** 2018-02-15

**Authors:** Kriti Kaushik, Ambily Sivadas, Shamsudheen Karuthedath Vellarikkal, Ankit Verma, Rijith Jayarajan, Satyaprakash Pandey, Tavprithesh Sethi, Souvik Maiti, Vinod Scaria, Sridhar Sivasubbu

**Affiliations:** 1 Genomics and Molecular Medicine, CSIR Institute of Genomics and Integrative Biology, Sukhdev Vihar, Mathura Road, New Delhi, 110025 India; 2 G.N. Ramachandran Knowledge Centre for Genome Informatics, CSIR Institute of Genomics and Integrative Biology, Sukhdev Vihar, Mathura Road, New Delhi, 110025 India; 3 Indraprastha Institute of Information Technology, Delhi, 110020 India; 4 Academy of Scientific and Innovative Research (AcSIR), New Delhi, 110025 India

**Keywords:** PARS, Zebrafish, Transcriptome, Gene regulation, RNA secondary structure

## Abstract

**Background:**

RNA is known to play diverse roles in gene regulation. The clues for this regulatory function of RNA are embedded in its ability to fold into intricate secondary and tertiary structure.

**Results:**

We report the transcriptome-wide RNA secondary structure in zebrafish at single nucleotide resolution using Parallel Analysis of RNA Structure (PARS). This study provides the secondary structure map of zebrafish coding and non-coding RNAs. The single nucleotide pairing probabilities of 54,083 distinct transcripts in the zebrafish genome were documented. We identified RNA secondary structural features embedded in functional units of zebrafish mRNAs. Translation start and stop sites were demarcated by weak structural signals. The coding regions were characterized by the three-nucleotide periodicity of secondary structure and display a codon base specific structural constrain. The splice sites of transcripts were also delineated by distinct signature signals. Relatively higher structural signals were observed at 3’ Untranslated Regions (UTRs) compared to Coding DNA Sequence (CDS) and 5’ UTRs. The 3′ ends of transcripts were also marked by unique structure signals. Secondary structural signals in long non-coding RNAs were also explored to better understand their molecular function.

**Conclusions:**

Our study presents the first PARS-enabled transcriptome-wide secondary structure map of zebrafish, which documents pairing probability of RNA at single nucleotide precision. Our findings open avenues for exploring structural features in zebrafish RNAs and their influence on gene expression.

**Electronic supplementary material:**

The online version of this article (10.1186/s12864-018-4497-0) contains supplementary material, which is available to authorized users.

## Background

RNA is a multitasking biomolecule, which not only acts as a messenger molecule to transfer genetic information from DNA to proteins, but also plays a vital role in regulation and catalysis of major biological reactions like transcription [1], post-transcriptional processing [2, 3] including splicing events, editing, degradation [4] and translation. In order to perform these processes, RNA adapts specific conformations owing to its ability to fold into secondary and tertiary structures [5]. The nucleotide sequence of RNA is primarily responsible for the secondary structure, formed by Watson Crick base pairing within the polynucleotide backbone. Subsequently, the tertiary structure is governed by the secondary structure and several other interactions with biomolecules [6]. The secondary structure of RNA is relatively stable and is present all throughout the length of mRNAs including CDS and UTRs [7]. A large number of diverse secondary structural motifs in mRNAs have been studied extensively including riboswitches, IRES [7], AU-rich, localisation elements [8] and structures that enhance transcription, alternative splicing and translation [2].

In addition to mRNAs, non-coding RNAs also display secondary structural features. The secondary structural elements in non-coding RNAs have been shown to regulate gene expression [9–12] and orchestrate the process of protein production. Small non-coding RNAs such as microRNAs [13] are known to fold in a pre-defined stem with loop structure that aids in binding to protein complexes and small molecules. Furthermore, long non-coding RNAs (lncRNAs) represent a class of regulatory RNAs that are abundantly present in eukaryotic transcriptome [14, 15]. LncRNAs display less nucleotide sequence conservation across species [16]; however, the secondary structural core of lncRNAs are conserved by reciprocal base pair mutations [17–22]. It is a well-known fact that structure and synteny conservation preserves the function of protein-coding mRNAs in species [23–27] separated by large evolutionary distances and this may also apply to non-coding RNAs. Therefore, understanding the secondary structure would be important for predicting the function of non-coding RNAs in general and lncRNAs in particular.

Zebrafish has been extensively used to study spatiotemporal expression profiles of genes including protein-coding genes [28, 29] and non-coding RNAs [30–32]. In recent years, several groups, including ours have documented spatiotemporal expression profiles of lncRNAs across early developmental stages [16, 33] and tissues in adult zebrafish [34, 35]. Amongst the lncRNAs discovered, only small fraction display nucleotide sequence conservation across different species. Majority of the lncRNAs do not display sequence conservation across evolutionary distances and this poses a significant hurdle for understanding their functional relevance. It is widely envisaged that the conserved structural features in non-coding RNAs especially lncRNAs may provide cues to conserved function across species [19, 22].

In this study, we probe the zebrafish transcriptome using Parallel Analysis of RNA Structure (PARS) [36, 37] to reveal the landscape of pairing probability at single nucleotide resolution. We undertook enzyme based probing of one day old zebrafish transcriptome using RNase V1 and S1 Nuclease to discover paired and unpaired nucleotide respectively. The enzyme cleaved fragments were subjected to next generation sequencing to yield pairing probability at single nucleotide resolution.

## Results

### Sequence data generation and mapping

About, 400 million reads were generated in total, with approximately 200 million reads in RNase V1 and S1 Nuclease cleaved samples respectively (Table 1).

**Table 1:** RNA-seq data production and alignment results for zebrafish poly (A) RNA reads

	S1 data (in millions)	V1 data (in millions)	Total (in millions)
Total Reads	213.05	204.96	418
Trimmed Reads	180.77	161.14	341.91
Total Mapped reads	169.67 (93.8%)	140.16 (86.9%)	309.84 (90.6%)
Uniquely mapped reads to genome	139.17 (76.9%)	104.85 (65.06%)	244.02 (71.37%)
Mapped reads to transcriptome	109.03 (60.32%)	59.73 (37.06%)	168.77 (49.36%)
Transcripts with load > 1	54,083		

The total number of sequence reads obtained from enzymatically probing (S1 Nuclease and RNase V1) the poly (A) RNA using RNA sequencing is mentioned. Mapped reads are aligned back to zebrafish genome (zv9)

The total sequencing reads generated from RNase V1 and S1 Nuclease cleaved fragments mapping to zebrafish transcriptome (*n* = 169 million) were aligned to 54,083 transcripts. Load score for all the transcripts were evaluated to check their abundance in the data. All the transcripts (*n* = 54,083) displayed a load ≥ 1 (Table 1).

**Fig. 1:**
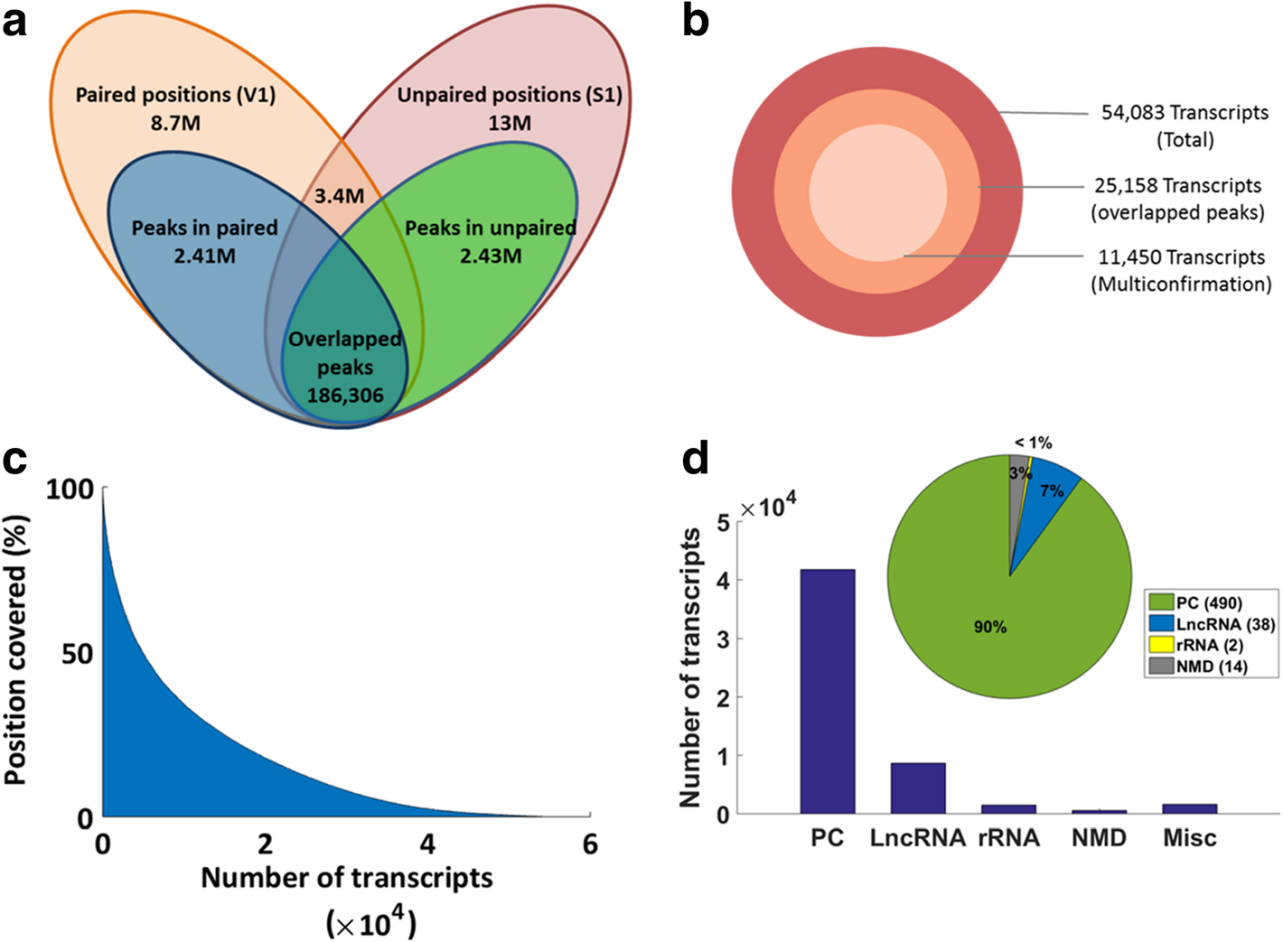
Overview of the transcriptome generated by PARS. **a**. Venn diagram representing paired and unpaired positions at 24 hpf zebrafish transcriptome obtained from PARS data. Approximately, 3.4 million positions are jointly obtained in both V1 and S1 cleaved samples. Blue (V1) and the green (S1) ellipse display positions with ratio score > 1, termed as peaks. Of these, 186,306 positions are overlapped peaks showing ambiguous positions. **b**. A total of 54,083 transcripts were assembled, from which 25,158 transcripts have positions with overlapping peaks in both V1 and S1 dataset. Amongst these 11,450 transcripts had more than 5 positions overlapping and are categorised as multi-conformation transcripts. **c**. Abundance of transcripts based on per base structure coverage. Coverage of the total transcripts (54,083) was estimated by the number of reads relative to the positions covered and the length of the transcript. Most of the transcripts (46,173) were less than 40% covered, 7366 transcripts were 40–85% covered, while 544 transcripts were > 85% covered. **d**. Bar plot representing the biotype of 54,083 transcripts. The biotype of the transcripts is represented. The inset pie shows distribution of transcripts with > 85% covered based on the function. PC: protein coding, lncRNA: long non-coding RNA, NMD: non-sense mediated decay, rRNA: ribosomal RNA, Misc: miscellaneous

To verify if the data obtained from the two datasets were unbiased, ratio score for each of the position was determined in both the samples (see Methods). The read counts for every position in the transcriptome were estimated in both RNase V1 (*n* = 8,700,581) and S1 nuclease (*n* = 13,151,051) cleaved samples (Fig. 1a). In total 18,375,999 unique positions were covered by both the enzyme cleaved samples in the transcriptome, of which 3,475,633 positions were jointly covered in both (RNase V1 and S1 Nuclease) datasets. There were 2,409,350 peaks (ratio score > 1) in RNase V1 dataset and 2,434,014 peaks in S1 Nuclease dataset. While, 186,306 positions had overlapped peaks (Fig. 1a) in both datasets i.e. 4% of the peaks exhibited ambiguous pairing probability, which suggested minimum biases in the data or impartial enzymatic cleavage and the two enzymes cleaved independent positions in the in-vitro folded zebrafish transcriptome.

PARS scores for all the respective positions (*n* = 18,375,999) cleaved by RNase V1 and S1 Nuclease were determined as per the formula described in Methods section. Normalisation constants K_v_ = 1.17 And K_s_ = 0.88 were used to normalise the read counts for every position.

The composite unique positions as obtained from RNase V1 and S1 Nuclease cleavage (*n* = 18,375,999) were aligned to 54,083 transcripts in zebrafish genome assembly (v79/Zv9). Amongst these transcripts, a total of 25,158 transcripts had positions represented by overlapping peaks in both the datasets with ratio score greater than one and 11,450 transcripts were termed as multi-conformation transcripts as they constituted at least five positions with overlapping peaks (Fig. 1b).

Position coverage i.e. positions with read starts across a transcript (generating from RNase V1/S1 Nuclease cleavages) was estimated. Out of 54,083 transcripts, 544 transcripts had more than 85% position represented by enzyme cleavage across the length of the transcript. While 7366 transcripts showed coverage from 40 to 85%, but a majority of transcripts 46,173 had coverage less than 40% (Fig. 1c). The biological distribution of 54,083 transcripts was determined. Majority of the transcripts (77%) were protein-coding, 16% were lncRNAs, 3% rRNAs, 1% NMD and rest 3% were labelled miscellaneous transcripts (Fig. 1d). Of these transcripts, those with more than 85% positions represented by enzyme cleavages were considered for further analysis. Further, the biotype of these transcripts is displayed in Fig. 1d with 90% of transcripts as protein coding, 7% lncRNAs, 3% NMD and less than 1% was rRNA.

### PARS-enabled pairing probability at single nucleotide resolution reveals structural conservation of protein coding genes across species

**Fig. 2:**
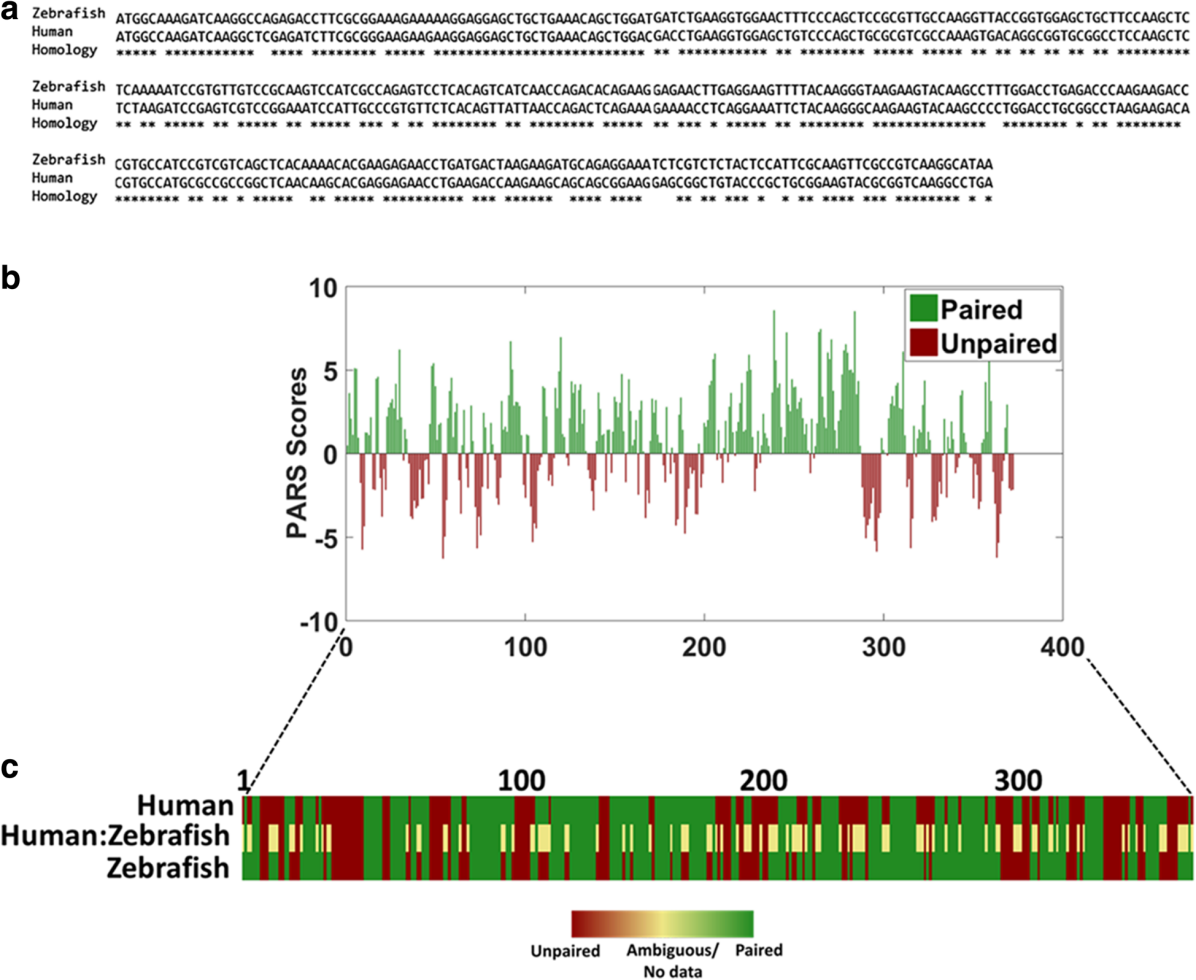
RNA secondary structure of *rpl35* in zebrafish and human. **a**. Sequence conservation within CDS of *rpl35* across human and zebrafish. *Rpl35* homologs in zebrafish and humans show 81% sequence homology. **b**. Bar graph representing PARS scores from CDS of *rpl35* in zebrafish. Red bars with negative PARS scores are unpaired, while positive scores in green are paired positions. **c**. Heatmap showing comparison of the paired and unpaired positions in *rpl35* of zebrafish and human. PARS based secondary structure signals reveal 71% structure homology. Red represents unpaired, green represents paired positions while yellow represents no consensus between the two homolog structures

The homologs of *rpl35* in zebrafish (NCBI Gene ID: 192,299) and human (NCBI Gene ID: 11,224) have 81% sequence homology in the CDS region encompassing 372 nucleotides (Fig. 2a). The investigation was restricted to CDS as UTRs have no sequence homology and are of varied lengths. Pairing probability for zebrafish *rpl35* was determined and plotted (Fig. 2b). Out of the 371 positions investigated, 233 are paired and 138 are unpaired positions. PARS scores of human *RPL35* were obtained from published PARS data of humans [37]. The comparison of PARS scores reveals 71% conservation in RNA secondary structure of CDS (Fig. 2c). Out of 360 positions, 71 positions are not conserved by sequence. However, of these 71 positions, 72% (*n* = 51) positions are structurally conserved.

### Comparison of PARS derived structure with enzymatic footprinting

Prior to analysing PARS-enabled pairing probability in zebrafish we wanted to investigate the validity of PARS using an orthogonal technology. We chose in vitro enzymatic footprinting to validate pairing probability of *ubiquitin c* (NCBI Gene ID: 777,766), a candidate protein-coding gene. *Ubiquitin c* was chosen as it had 97% nucleotide positions represented by enzyme cleavage across the length of the transcript. Since UTRs are known to play important role in gene regulation owing to the secondary structural features [38], we chose the 3’ UTR of *ubc* for validation. In addition, short length of 105 bases was considered favourable for enzymatic footprinting.

**Fig. 3:**
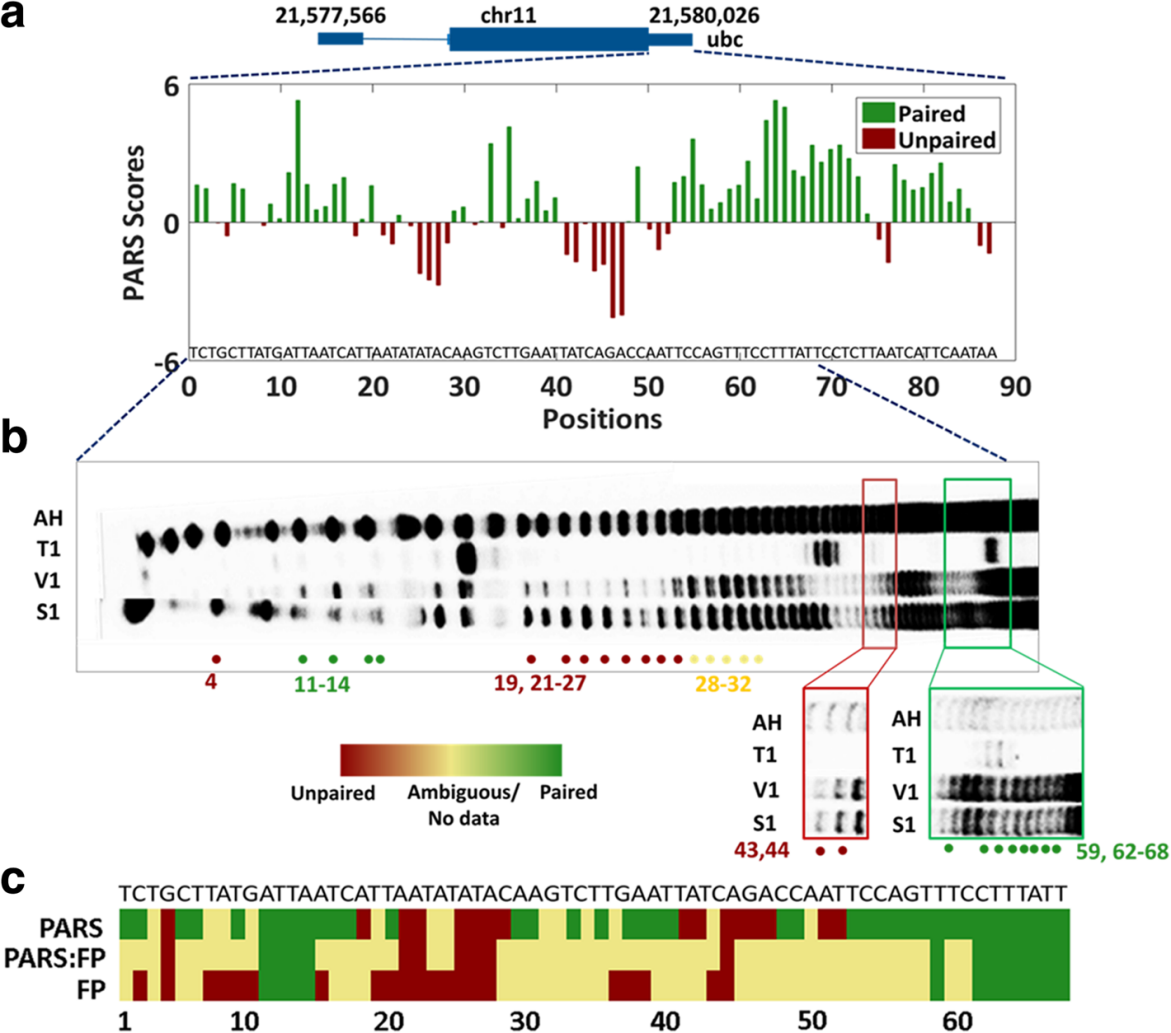
Comparison of RNA structures of *ubc* 3’UTR as determined by PARS based pairing probability and enzymatic footprinting using RNase V1 and S1 Nuclease. **a**. Bar plot represents PARS scores of 3’UTR region of *ubiquitin c (ubc)*. Out of 105 positions, 87 positions are captured by PARS. **b**. Enzymatic footprinting of *ubc* 3’UTR probed by S1 Nuclease and RNase V1. Nucleotide positions are correlated with alkaline hydrolysis (AH) ladder and RNase T1 (G) ladder. Positions with similar structural pattern with PARS scores are highlighted. Red dots indicate unpaired positions; green indicates paired positions while yellow represents ambiguous regions. **c**. Heatmap representing secondary structure of 68 positions of *ubc* 3’UTR as determined by PARS and enzymatic footprinting (FP). Top panel represents PARS pairing probability; bottom panel indicates enzymatic footprinting pairing probability; middle panel represents the consensus between the two (PARS: FP). Red represents unpaired, green represents paired and yellow represents ambiguous regions

Out of the 105 positions investigated in the *ubc* 3’UTR, PARS scores were obtained for the first 87 positions. PARS scores could not be obtained for the last few positions, due to low quality reads obtained from enzyme cleaved fragments at the ends of the transcripts. PARS signals for *ubc* 3’UTR are displayed in Fig. 3a for the 87 nucleotide positions of which 59 are paired, 27 are unpaired and one has no PARS score.

Enzymatic probing followed by gel based footprinting of the *ubc* 3’UTR revealed differentially cleaved nucleotide positions (Fig. 3b). Only 40 positions were resolved in one gel. Higher molecular weight positions up to 68th nucleotide positions were resolved in separate gels. Relative structure signals for every nucleotide position as procured from enzymatic footprinting, PARS and the consensus between the two methods are represented as a heatmap (Fig. 3c). Out of the 68 positions resolved in footprinting gel, 28 positions (41%) match the pairing and unpairing possibilities as covered by PARS scores. In summary, conservative estimates of pairing probabilities as determined by PARS and enzymatic footprinting displayed modest concordance with each other.

### Attributes of RNA secondary structure as determined by PARS based pairing probability across functional units of mRNAs

**Fig. 4:**
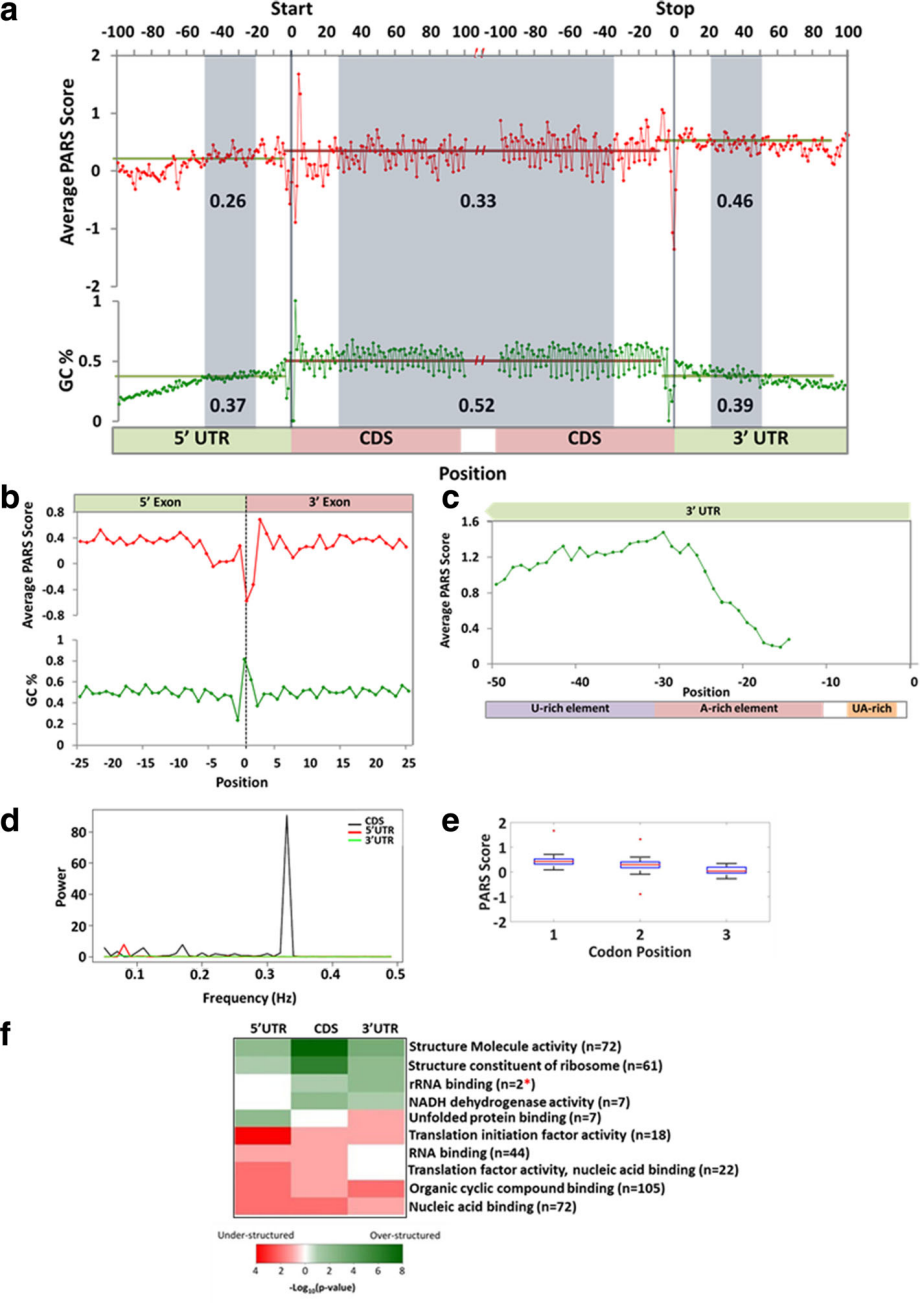
PARS reveals distinct RNA secondary structural signatures in functional units of transcripts. **a**. PARS scores across the 5’UTR, the coding region (CDS), and the 3’UTR of Zebrafish mRNAs are represented. PARS scores averaged across 451 transcripts with load > 1 and position coverage > 85%, aligned by the translational start and stop sites are represented. Averaged PARS scores and GC% are reported for regions are shaded in grey. **b**. Line graph representing average PARS scores and GC% across 25 nucleotides flanking the splice junctions of 451 transcripts are represented. **c**. Line graph displaying average PARS scores for last 50 nucleotides of the 3’ UTRs (*n* = 451) are represented. **d**. Line graph representing amplitude vs frequency of the Discrete Fourier Transform analysis of the average PARS scores of CDS, 3’ UTR and 5’ UTR corresponds to 451 transcripts. The highest frequency peak is obtained at 0.33 in CDS, showing a periodicity of 3 bases. **e**. Boxplot for average PARS scores for every codon position for first 100 CDS positions in 451 transcripts. The pairing probability of every position in a codon follows 1 > 2 > 3 (*p* value = 1.9e-07). Every position significantly differs from the other position by a *p* value = 1.702e-08 (ANOVA). **f**. Region-wise pattern of RNA secondary structures within enriched molecular function GO categories. The heatmap represents the region-wise (5’UTR, CDS and 3’UTR) significant *p*-values obtained from Wilcoxon rank sum test performed using the average PARS scores calculated for transcripts belonging to each enriched GO category. Red color suggests that genes belonging to the specific GO category shows under-structuring or lower PARS scores than the expected average PARS score for the region, where as shades of green depict over-structuring of genes belonging to the specific GO category in the respective regions. The asterisk * indicates that no significant conclusion can be drawn for a small number of genes (*n* = 2) in rRNA binding category

The nature and extent of RNA secondary structure along different regions of spliced mRNAs, namely coding region (CDS), transcription start and stop sites, splice sites, 5′-untranslated region (5’-UTR), 3′-untranslated region (3’-UTR) and poly-A sites were compared. In order to study this, the protein coding transcripts with at least 85% read start positions across the transcript length were prioritised. Further, amongst these transcripts, only those with well annotated translation start and stop signals were selected. Nucleotide position-wise average PARS scores for each region were calculated and results were plotted (Fig. 4). Amongst the transcripts with at least 85% read start positions (*n* = 544), there were 451 transcripts with well-defined translation start and stop sites. Average PARS score was 0.33 in CDS, 0.26 in 5’UTR and 0.46 in 3’UTR. A sharp decrease in pairing probabilities of nucleotide positions was seen at the translational start (*p-*value = 1.83 × 10^− 7^) and stop sites (*p-*value = 8.44 × 10^− 57^) (Fig. 4a). A sharp increase in the pairing probability was followed after the translation start sites (*p-*value = 5.37 × 10^− 36^). However, the 3’-UTR was highly structured followed by CDS and 5’-UTR. The structure signals at 5’UTR were positively correlated with GC content (*r* = 0.32), but showed negative correlation at CDS (*r* = − 0.4) and no correlation at 3’UTR (*r* = − 0.003). A periodic pattern of pairing probability was also observed in CDS, but absent in UTRs.

We also explored the pairing probability across splice sites of highly expressed mRNAs. The splice junctions (*n* = 2538) across the 451 transcripts were aligned. Average pairing probabilities of 25 nucleotide positions flanking the splice junctions were calculated (Fig. 4b). It was observed that the pairing probability of terminal dinucleotide at the 5′ exon are different relative to the rest of the positions of the transcript (*p-*value = 5.5 × 10^− 28^). Similarly, the pairing probability of the first dinucleotide at 3′ exon are different relative to the rest of the positions of the transcript (*p-*value = 1 × 10^− 3^). However, a comparison of the dinucleotides at the splice junctions displayed that terminal dinucleotides at 5′ exon are structurally flexible than first dinucleotides at 3′ exon (*p-*value = 2.5 × 10^− 5^). This pattern of secondary structure signal at the splice junctions is inversely correlated with GC content.

Additionally, we investigated the pairing probability across poly-A sites at 3’UTR across mRNAs. The transcripts (*n* = 451) were aligned at ends of 3’UTRs of transcripts and average pairing probability till 50 positions upstream were calculated (Fig. 4c). Positions from − 10 to − 30 showed low structure signals (*p-*value = 4.5 × 10^− 18^) relative to upstream region from − 30 to − 50.

The periodic structural pattern in CDS as observed in Fig. 4a was further investigated. Therefore, to decipher if the primary sequence codes for a structural pattern, periodicity of pairing probability in the CDS was tested. The periodicity was determined on first 100 CDS positions, first 100 3’-UTR and last 100 5’-UTR positions from average pairing probabilities of 451 transcripts. The highest amplitude was observed at a frequency of 0.33 i.e. periodicity of 3 bases in the CDS region. However, the UTRs have very low amplitude relative to CDS and no periodicity was seen at 3 bases as shown in Fig. 4d. When the CDS positions were binned in codons, every position in a codon had pairing probability significantly different from the other two positions (*p-value =* 1.702 × 10^− 8^*)* (Fig. 4e). The first position had the highest pairing probability compared to the second position, while third position had the least ability to be present in a paired conformation (*p-*value = 1.9 × 10^− 7^). This is repeated in a cycle of three, suggesting that similar to primary sequence, pairing probabilities in a codon also display a pattern.

Furthermore, these 451 transcripts were categorised in 40 classes of gene ontology based on their molecular function to survey any similar structural features within the same Gene Ontology (GO) class. Out of forty, only ten classes showed significantly similar pattern of pairing probability within a category. A heatmap of these ten classes in Fig. 4f displays four classes, which are over structured with respect to the mean PARS scores of the total 451 transcripts. Genes with ‘structural molecule activity’, ‘structural constituent of ribosomes’ and ‘NADH dehydrogenase activity’ are highly structured in CDS relative to UTRs. However, ‘rRNA binding’ genes have higher structure in 3’UTR than CDS. On the contrary, ‘translation factors’ and ‘organic cycle compound binding’ have negligible secondary structural features in UTRs compared to CDS. Genes with ‘nucleic acid binding’ have lower pairing probability in CDS and 5’UTR than 3’ UTR. Secondary structures were present in 5’UTR region of genes with ‘unfolded protein activity’ with single stranded features in 3’UTR. Thus, the ten classes of gene ontology displayed similar secondary structural pattern amongst transcripts of the same group, across CDS and UTRs.

### PARS-enabled pairing probability at single nucleotide resolution reveals secondary structures of candidate non-coding RNAs in zebrafish

**Fig. 5:**
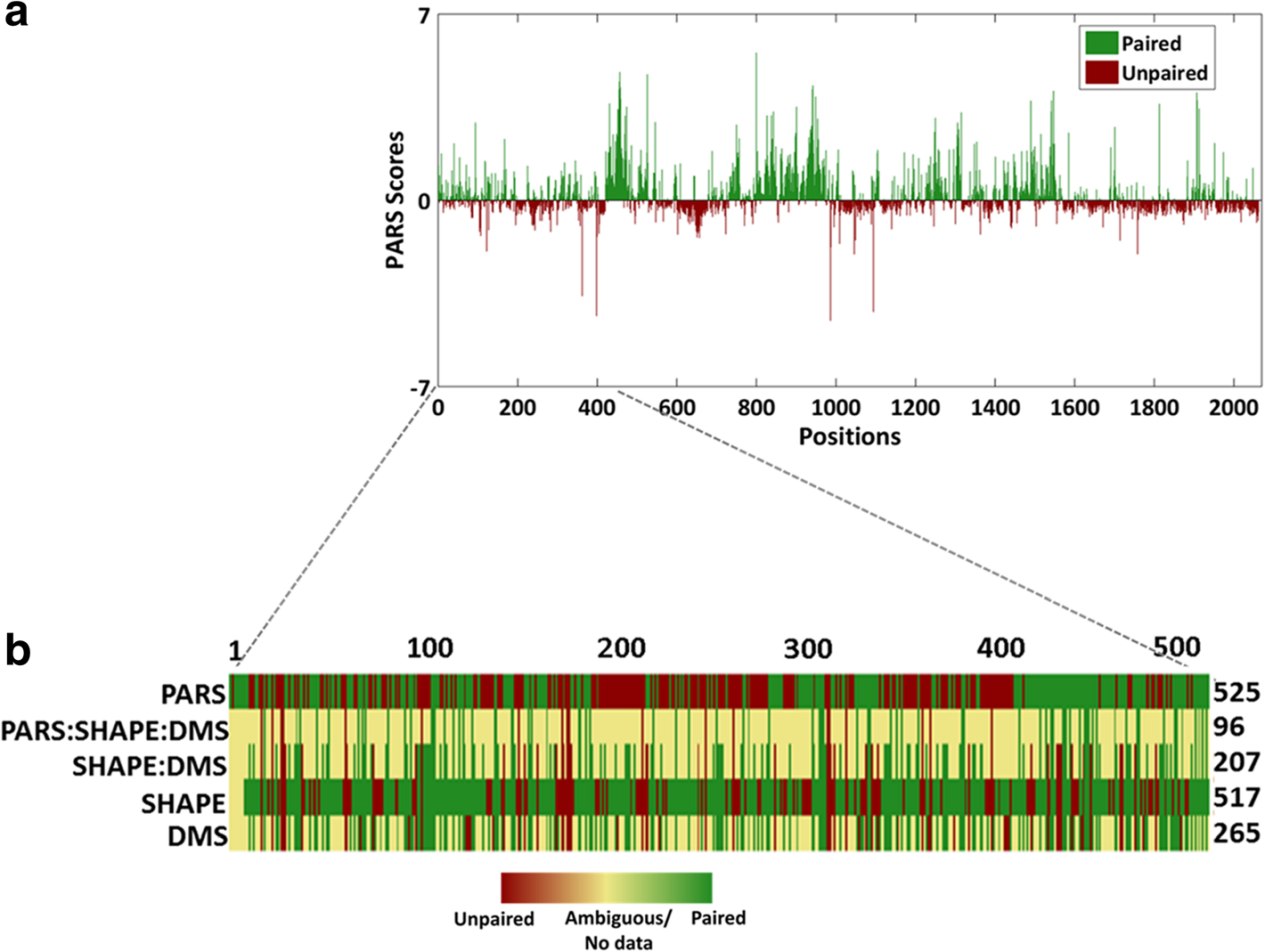
Secondary structure of human non-coding RNA, *HOTAIR.* **a**. Bar graph representing PARS scores of 2062 positions of *HOTAIR*. Red bars with negative PARS scores are unpaired, while positive scores in green are paired positions. **b**. Heatmap with comparison of the paired and unpaired positions in domain 1 (1 to 525 positions) of *HOTAIR*. PARS scores are compared with structure data obtained from SHAPE and DMS probing. Red represents unpaired, green represents paired positions while yellow represents no data for that position. Upon comparison, 207 out of 518 positions were correlated by SHAPE and DMS, while PARS has consensus of 96 positions amongst these 207 positions

After deciphering the secondary structure pattern across functional units of mRNAs, we utilised PARS to determine the structures of non-coding RNAs. The efficiency of PARS, an enzyme based probing method was evaluated by comparing with structures derived from chemical probing methods for non-coding RNAs. The human lncRNA, *HOTAIR* was used as a positive control to validate PARS scores for non-coding RNAs. Approximately, 2.2 kb long *HOTAIR* was structure probed with RNase V1 and S1 nuclease to obtain PARS scores for 2039 positions. These scores were plotted and are illustrated in Fig. 5a. In recent years, *HOTAIR* structure has been elucidated by three different methods namely, SHAPE (Selective 2′-hydroxyl acylation analysed by primer extension), DMS (Dimethyl sulfate) probing and Terbium chloride probing [19]. PARS structure was compared with SHAPE and DMS derived structure (Fig. 5b) for one of the domains (Domain I) of *HOTAIR*. The three techniques compared here have different mechanisms namely, PARS is enzyme based method, while SHAPE and DMS are chemical probing methods. PARS scores were obtained for all 525 positions in domain I of *HOTAIR*, while SHAPE captured 518 positions and 265 positions were obtained from DMS probing. Pairing probabilities as determined from SHAPE and DMS have 207 positions in consensus with each other. Amongst these 207 positions, PARS has 47% positions in consensus with SHAPE and DMS based methods (Fig. 5b).

**Fig. 6:**
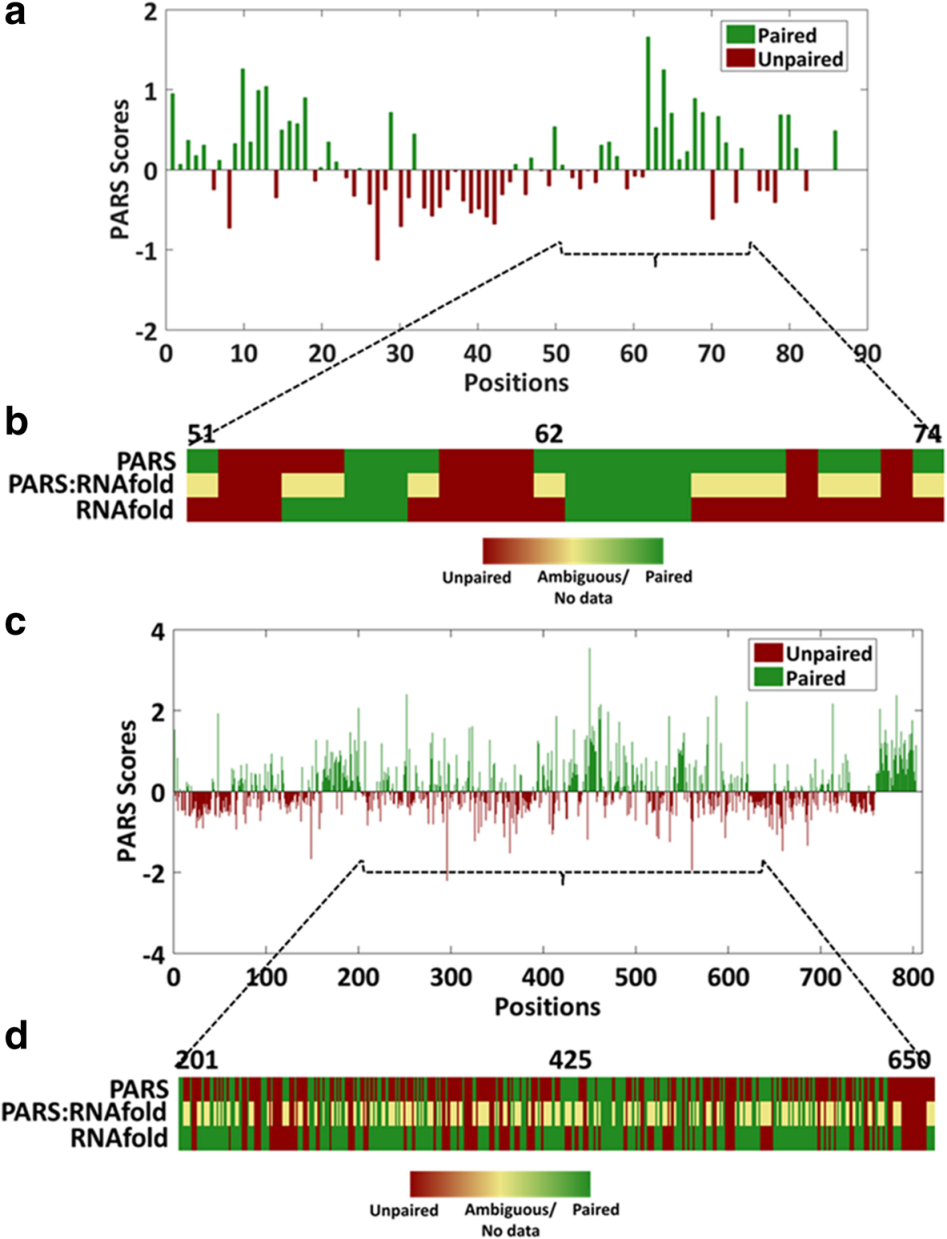
Secondary structure of zebrafish non-coding RNA as determined by PARS. **a**. Bar plot representing PARS scores of *y-rna* for 83 positions out of 106 positions. **b**. Heatmap with comparisons of pairing probability of the binding region of *y-rna* as determined by PARS and computational predictions by RNAfold. **c**. Bar plot representing PARS scores of *tie1-as* for 803 positions out of 819 positions. **d**. Heatmap with comparison of pairing probability of *tie1-as* as determined by PARS and RNAfold

Having determined the single nucleotide pairing probability of *HOTAIR*, next we investigated secondary structure of the candidate non-coding RNAs in zebrafish, evolutionary conserved *y-rna* and *tie1-as* (antisense lncRNA to tyrosine kinase containing immunoglobulin and epidermal growth factor homology domain-1). Pairing probabilities of these non-coding RNAs were resolved using PARS to correlate the secondary structural patterns with their functional properties. *y-rnas* are non-coding transcripts of 106 bases in zebrafish and are evolutionary conserved in vertebrates (human, *Xenopus* and zebrafish) [39]. They regulate the initiation of DNA replication after mid-blastula transition by associating with origin recognition complex and factors like CDT1 [39]. Positions 51–75 are regulatory regions that are involved in interaction with other partners. Pairing probability at single nucleotide resolution will ease the better understanding of the role of zebrafish *y-rna* as displayed in Fig. 6a with the regulatory nucleotide positions highlighted. PARS captured 83 positions out of 106, of which 40 positions are unpaired and 43 positions as paired. Furthermore, the RNAfold predicted structure of *y-rna* (Fig. 6b) highlights 58% concordance with PARS derived secondary structure in the regulatory region.

*Tie1-as*, is an antisense lncRNA to the protein coding gene *tyrosine kinase containing immunoglobulin and epidermal growth factor homology domain-1 (tie-1)* [40]. Like several other antisense RNAs, *tie-1as* also binds to *tie-1* RNA and regulates its levels. Keguo and co-workers have shown the hybrid structure of *tie1* and *tie1-as* by computational predictions. PARS assisted structure probing at single nucleotide resolution (Fig. 6c) may aid in better understanding of this hybrid. Out of 819 positions of *tie1-as*, 803 are captured by PARS assay. Of these, 449 positions are unpaired and 354 positions are paired. The RNAfold predicted structure of *tie1-as* shows 53% concordance with PARS assisted structure in the binding region of this lncRNA (Fig. 6d).

### Transcriptome-wide single nucleotide resolved secondary structure map of zebrafish

**Fig. 7:**
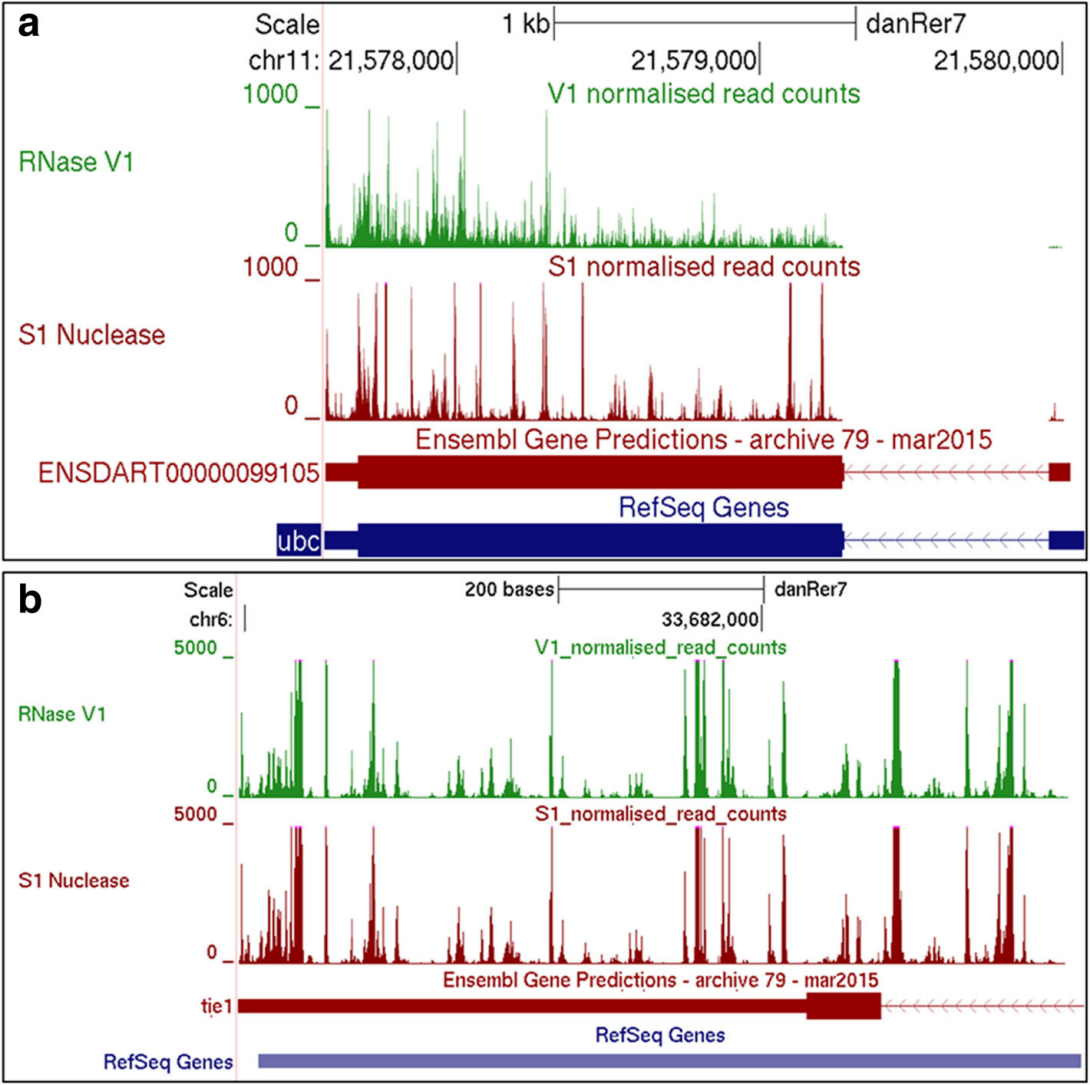
UCSC snapshot of single nucleotide resolved RNA secondary structure map for (**a**) *ubiquitin c* and (**b**) *tie1-as*

We have developed a web based online resource that provides pairing probabilities of zebrafish transcriptome at single nucleotide resolution. The normalised read start counts for every position generated from RNase V1 and S1 Nuclease catalysed fragments in the genome has been provided as bigwig files. This can be uploaded on UCSC genome browser (http://genome.ucsc.edu/cgi-bin/hgTracks?db=danRer7&hubUrl=http://genome.igib.res.in/upload_files/zf_pars_hub/hub.txt) under zv9 assembly. A snapshot of *ubiquitin c* (Fig. 7a) and *tie1-as* (Fig. 7b), displays an example of pairing probabilities of zebrafish transcriptome (Additional files 2, 3, 4, 5, 6, 7, 8 and 9).

## Discussion

Multiple genome scale sequencing projects have highlighted that a large proportion of the genome actively contributes towards the transcriptome, most of which is engaged in regulatory activities. In order to gain insights into the functional aspect of the regulatory transcriptome, it is important to understand their ability to interact with other biomolecules by the virtue of their structure. The primary information for intramolecular pairing is embedded in the RNA sequence [37]. The Watson-Crick base pair driven secondary structure thereby provides a template for the RNA to fold upon itself and sets the stage for long range interactions. Therefore, it is important to uncover the hidden layer of information in the RNA secondary structure, to further understand the tertiary interactions. Conventional gel based methods of assessing RNA secondary structure focused on single RNA species in isolation. However, it is well-known that the structures are influenced in presence of a heterogeneous pool of transcripts. Lately, several RNA probing methods [36, 37, 41–44] are coupled with high throughput sequencing to decipher the transcriptome-wide secondary structure in diverse organisms. Amongst these, PARS has been applied to yeast and human transcriptomes to determine pairing probability at single nucleotide resolution. Zebrafish has emerged as a model organism to study various biological processes including human diseases. Understanding RNA secondary structure organisation and its features across functional units of transcripts would provide insights into the functioning of transcripts in zebrafish.

Pairing probabilities obtained from RNase V1 and S1 Nuclease cleavage of zebrafish transcriptome suggested that the transcriptome was equally paired and unpaired. However, the structure maps of other eukaryotic transcriptome such as, *A. thaliana* [45] revealed that a larger part of the transcriptome was unpaired. In the zebrafish transcriptome, the RNase V1 and S1 Nuclease enabled cleavages displayed distinct signatures with only 4% overlap of the structure signals, hinting to the ambiguous nature of the pairing probabilities at these nucleotides. Previously, structure profiling of the yeast transcriptome reported 7% nucleotide positions [36], while that of human transcriptome constituted 3.7% nucleotide positions to have ambiguous pairing probability respectively [37].

Out of the total transcripts (*n* = 54,083), a majority (77%) mapped to protein coding genes, while lncRNAs were relatively low (16%). This could be possible as lncRNAs possess a lower expression than protein-coding genes, and hence are not sequenced at a greater depth. This was similar to findings from PARS probed human transcriptome [37].

The well-known protein-coding gene (*rpl35)* in zebrafish was found to possess sequence and structure homology with its human orthologue *Rpl35*. We also studied another conserved gene - nucleoplasmin 1a (npm1a, 852 bases, ZFIN:ZDB-GENE-021028-1). This gene showed 62% sequence homology in the CDS region with its human ortholog NPM (NCBI ID: 4869). We observed an overall structure conservation of 329 bases (38%) out of which 197 positions (60%) were also conserved at the sequence level. In summary, we observed that although *npm1a* has similar sequence conservation as *rpl35* with its human ortholog, the structure conservation does not follow any specific trend. Given that the extent of over-structuring or under-structuring is enriched for specific gene ontology categories in a species as shown in Fig. 4f, the same might hold true across species and may not always be simply a function of the sequence conservation.

Regulation of gene expression at post-transcriptional and translational levels is governed by the functional units of mRNA such as translation start and stop sites, CDS, splice sites, UTRs and poly-adenylation sites. Distinct RNA secondary structure features in zebrafish transcriptome were observed corresponding to the different functional units of highly expressed protein coding transcripts. A sharp decrease in the PARS scores was noticed at translational start and stop signals. This may suggest ribosome accessibility to coding regions and initiation of translation. Similar features were also observed in other transcriptomes studied such as humans [37], yeast [36] and Arabidopsis [43]. This is in accordance to the presence of IRES (internal ribosome entry sites) present at translational start signals in eukaryotes [46] which are structured elements. As observed in our study, structure signals with low pairing probability at translational stop sites were also reported in humans [37] and yeast [36].

Zebrafish CDS region had higher pairing probability than 5’-UTRs but displays lesser pairing probability than 3’-UTRs. However, structure signals in the CDS of yeast transcriptome showed higher pairing probability compared to UTRs [36]. The three base periodicity observed in the CDS region of zebrafish transcriptome was correlated to that observed in yeast [36] and Arabidopsis [43] suggesting a common universal regulatory feature in translating regions in eukaryotes. This was similar to the three base sequence periodicity in coding exons of DNA [47]. There have been reports suggesting (RNY)_n_ sequence periodicity in CDS of various genomes. The pattern of structure signals within a zebrafish codon was also consistent, such that every first base in a codon had the highest pairing probability suggesting structural constraints relative to the second base, while the last base has the lowest pairing probability suggesting structural flexibility. The structural constraint observed in the first position of the codon in zebrafish mRNAs might create a steric hindrance so that the subsequent codon positions have more steric flexibility. This is in contrast to what Kertesz et al. reported within the yeast, where the second base of the codon had highest pairing probability followed by third and first. The triplet periodicity in the protein coding regions of transcript suggests the translational efficiency of the transcripts [43]. In our study, we observed a three base structure periodicity in the CDS, suggesting a righteous conformation for the occupancy of ribosomes. Additionally, as periodic structure signals are distinct in coding and non-coding UTRs, the structure signals can be employed to annotate the unknown regions in the zebrafish transcriptome.

Distinct structure signals were observed at the splice site junctions of the zebrafish transcriptome. The dinucleotides at the end of the 5’exon possess structure signals with low pairing probability and first dinucleotides of 3’exon have structure signals with higher pairing probability relative to the rest of the positions in a transcript. This was similar to the findings observed at splice junctions of mRNAs in human transcriptome [37].

The 5’UTR regions in zebrafish mRNAs on average possess lowest pairing probability compared to CDS and 3’-UTRs. This is also true for yeast [36], Arabidopsis (in vivo) [43] and mouse (in silico) [48]. The 3’UTR regions display higher pairing probability compared to CDS on average, as 3’UTRs constitute several regulatory elements. Albeit, GC% of the bases rules the structure signals, the UTRs in zebrafish have lower GC content than CDS. Similar to the findings in humans, a low consensus between GC content and RNA secondary structure signals was observed [37]. Moreover, the analysis of structure signals at poly-A sites revealed that *A-rich elements* (− 10 to − 30 nt) [49–51] endure structure signals with lower pairing probability relative to *upstream stimulating element* (USE), suggesting the accessibility of *Cleavage and Polyadenylation Specificity Factor* (CPSF) protein [51].

In extension to this, when the highly expressed protein coding transcripts were grouped on the basis of their molecular function, genes with functions of ‘structure molecule activity’ and ‘structural constituent’ of ribosomes such as rRNA had higher pairing probabilities across CDS and UTRs. While, genes with ‘translation factor activity’ had lower pairing probability in CDS than UTRs as they need to be actively translated and highly regulated by domains in UTRs. The presence of structured elements (high pairing probability) at 5’-UTRs signify regulation at translation level, whereas structures at 3’-UTRs represent post-transcriptional processing. Similarly, structure profiling of yeast transcriptome [36] and Arabidopsis transcriptome [43] revealed correlation between RNA secondary structure signals and biological function of mRNAs.

PARS scores generated for zebrafish non-coding RNAs were endorsed using known structure of human *HOTAIR* determined by chemically probing using SHAPE and DMS [19]. PARS scores showed positive correlation with the other two derived structures, thereby confirming the broad utility of PARS for determining secondary structure of non-coding RNAs. The difference in consensus between PARS and the other two techniques could be due to the usage of different structure sensitive reagents in these studies. Nucleases possess steric hindrance in catalysing large structured elements. The efficiency of RNase V1 is limited by helix length whereas chemical probing reagents are much more specific due to smaller size. However, chemicals such as DMS can probe only unpaired adenine or cytosine, therefore not providing information about uracil or guanine. SHAPE, utilises reagents that interact with sterically flexible nucleotides, but can be carried out only for known sequences. In comparison, PARS an enzyme-based probing method, provides structure signals for every position in the transcript.

In the recent few years, non-coding RNAs especially lncRNAs have been extensively studied in zebrafish cataloguing lncRNA transcripts expressed in different developmental stages [16, 33] and adult tissues [34]. Of these, 13–35% lncRNAs are overlapping to protein coding genes in sense or antisense direction [35]. One of the earliest studied lncRNA in zebrafish was *tie1-as*, antisense to *tyrosine kinase containing immunoglobulin and epidermal growth factor homology domain-1 (tie-1)* [40]. Similarly, *y-rna* is a small non-coding RNA, which interacts with DNA replication machinery at maternal to zygotic transition. Both of these non-coding transcripts play a pivotal role in the developmental stages of zebrafish. Therefore, in order to aid in investigating the function and binding partners of the transcripts and the mechanism of regulation, the secondary structural trends in these transcripts were visualised using PARS. This presents the first experimentally validated structures of non-coding RNAs in zebrafish. Furthermore, the single nucleotide resolved pairing probability map of zebrafish transcriptome could be evaluated to predict miRNA binding sites. Strong AGO binding sites display lower pairing probabilities at − 1 to 3 nt upstream of miRNA target sites [37]. Pairing probabilities at the UTRs of transcripts can be availed to verify the miRNA target sites.

In addition, the single nucleotide pairing probabilities could also be utilised to identify riboSNitches [37], which are secondary structure elements that change in the presence of single nucleotide variations. Previous studies have documented approximately 15 million Single Nucleotide Variations (SNVs) in different strains of zebrafish [52, 53]. The regulation of gene expression by riboSNitches could be evaluated by studying the structure signals across these SNVs, which could provide insights on how SNVs contribute to modulating gene expression.

## Conclusion

We present the first PARS-enabled secondary structure transcriptome map of zebrafish, which documents pairing probability of RNA at single nucleotide precision. This has facilitated the identification of unique structural patterns across functional units of mRNA. We also present the enzyme probed structures of selected regions of candidate non-coding RNAs such as *tie1-as* and *y-rna* in zebrafish. This study is not without limitation. Currently, PARS has been executed by folding the transcripts in-vitro with the consensus that most of the structure signals are embedded in the sequence. However, future studies may be carried out on native de-proteinised transcripts to see the extent to which in vivo structures deviate from the present ones. The technique can be further explored to determine RNA structure of full length candidate lncRNAs in zebrafish. This study provides a basal data to plan experiments for long range intra- and inter-molecular interactions of transcripts using Psoralen Analysis of RNA Interactions and Structures (PARIS) [54]. This transcriptome-wide secondary structure map at single nucleotide resolution adds to the ever increasing genomics resources for zebrafish and would aid in improving our understanding of the zebrafish transcriptome.

## Methods

**Fig. 8:**
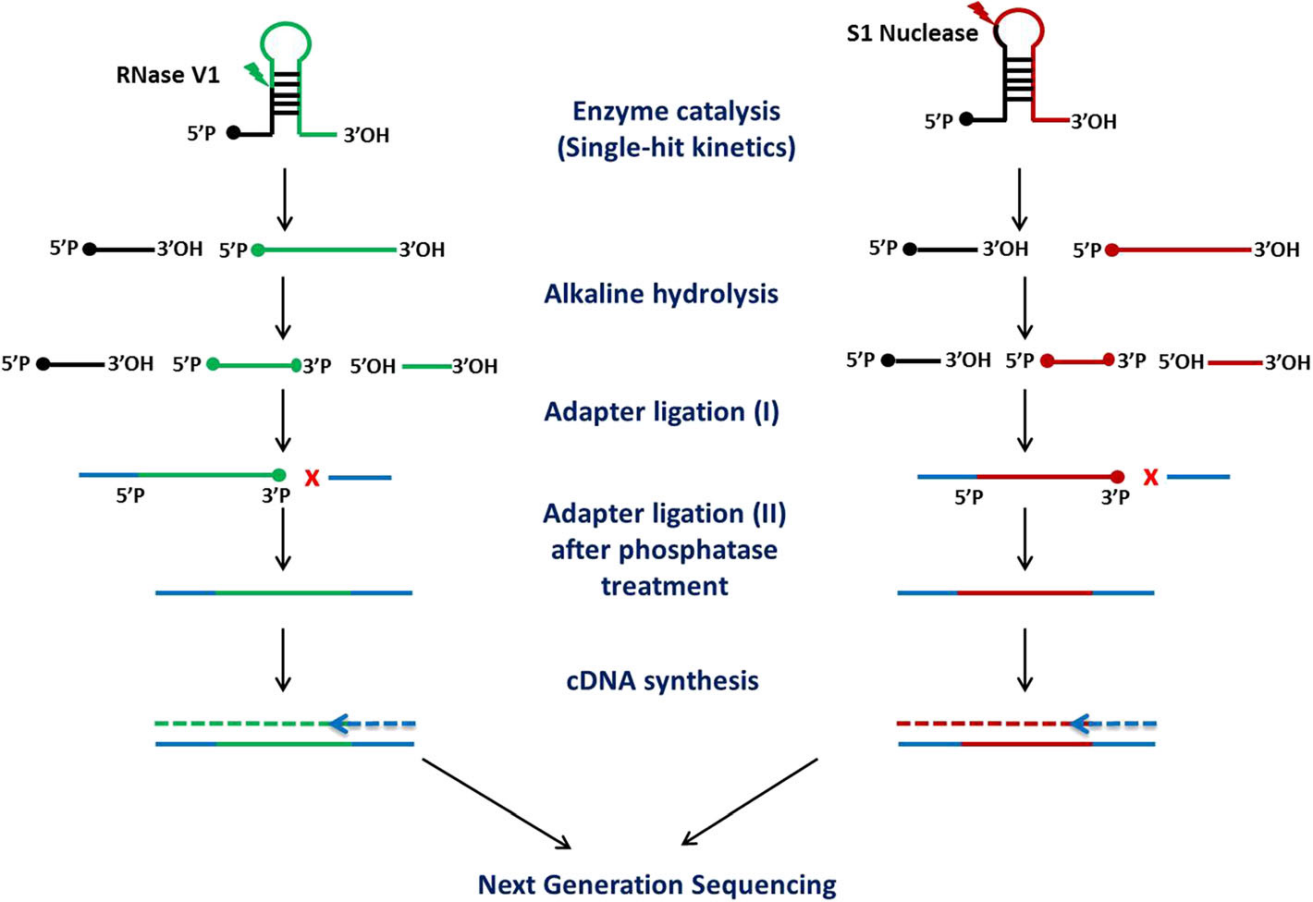
Schematic of RNA structure probing by PARS in zebrafish. Poly-A RNA from zebrafish is folded in-vitro. The folded RNA is cleaved by RNase V1 and S1 nuclease separately. The enzyme cut sites generate 5’P ends and 3’ OH ends at the cleaved sites. Long fragments generated by single-hit kinetics are further fragmented by alkaline hydrolysis, which blocks the 3′ site of the enzyme-cut fragments. Sequencing adapters are ligated to the 5′ end followed by alkaline phosphatase treatment to 3’ P group. Adapters are ligated to 3’ends followed cDNA synthesis and PCR purification of the library. Appropriate size of the library is maintained by purification by nucleic acid beads. Sequenced reads are aligned back to the genome and only unique reads with the correct read start positions are considered for PARS score calculation

Transcriptome-wide RNA secondary structure profiles in developing zebrafish was captured using Parallel Analysis of RNA Structure (PARS) [36, 37]. The transcriptome from one-day-old (24 hpf) zebrafish embryos was subjected to enzymatic cleavage by RNase V1 and S1 nuclease catalysis (Additional file 1: Figure S1). The enzyme-based cleavage reaction was tightly regulated to derive single-hit kinetics. In-vitro reconstituted zebrafish transcriptome was subjected to cleavage by RNase V1 at 0.000125 U for 45 s (s) and by S1 Nuclease cleaved at 10,000 U for 10 min (min) respectively in RNA structure buffer (1X). The enzyme cleaved fragments were adapter ligated and processed for next generation sequencing using semiconductor based chemistry on Ion Proton Platform as described below. A detailed schematic of Parallel Analysis of RNA Structure is shown in Fig. 8.

### RNA isolation

Assam wild type (ASWT) strains of zebrafish maintained at CSIR-Institute of Genomics and Integrative Biology were used in this study [52]. One-day old zebrafish embryos were collected in the eppendorf tubes and water was decanted. The vials were immediately snap frozen at − 80 °C and processed for RNA isolation. Approximately, 200 μg of total RNA was isolated from 24 hpf ASWT zebrafish (*n* = 250) using RNeasy kit (Qiagen, USA) as previously described [55]. Poly-A RNA was enriched using oligo-dT Dynabeads (Life Technologies, USA) using manufacturer’s protocol to yield 4 μg of processed transcripts from the initial pool. RNA pool of Poly-A transcripts was divided into two parts (2 μg each) for individual catalysis by RNase V1 (Life Technologies, USA) and S1 nuclease (Thermo Fisher Scientific, USA). Likewise, five biological replicates were put using 1250 embryos in total.

### Enzymatic probing of RNA

The resulting RNA pool was processed for PARS heated at 90 °C for 2 min followed by snap-chilling in ice (at 0–4 °C). Further, the poly-A pool was folded in RNA Structure Buffer (Life Technologies, USA) containing 10 mM Tris pH 7, 100 mM KCl, and 10 mM MgCl_2_ slowly from 4 °C to 28 °C for 25 min. Each 2 μg of Poly-A RNA was digested with 10 μl (0.000125 U) of RNase V1 (Life Technologies, USA) for 45 s and 10 μl (10,000 U) of S1 Nuclease (Thermo Fisher Scientific, USA) for 10 min to achieve single hit kinetics. Enzyme-cleaved fragments were further purified using equal volume (100 μl) of phenol: chloroform: isoamyl alcohol (Invitrogen, USA) at 13,000 rpm (4 °C) for 10 min. RNase V1 cleaved RNA pool in the top aqueous layer was extracted and 20 μl of Inactivation/Precipitation buffer (Life Technologies, USA) was added, followed by 1 h (h) incubation at -80 °C. Further, 20 μl of 3 M sodium acetate, 1 μl of glycogen and 300 μl of cold ethanol was added to precipitate RNA at -80 °C for 1 h. S1 Nuclease cleaved RNA pool in the top aqueous layer was extracted and 20 μl of 3 M sodium acetate, 1 μl of glycogen and 300 μl of cold ethanol was added to precipitate RNA at -80 °C for 1 h. Further, the purified RNase V1/S1 Nuclease digested samples were fragmented by alkaline hydrolysis buffer (Life Technologies, USA) containing 500 mM Sodium bicarbonate at 95 °C, for 1.5 min which generated 3’phosphate groups at the enzyme cleaved fragments. The reaction was stopped using 2 μl of 3 M sodium acetate, and further precipitated using 2 μl of glycogen and 300 μl of 100% ethanol at -80 °C for 3–4 h. A detailed schematic of Parallel Analysis of RNA Structure is shown in Fig. 8.

### Preparation of library and sequencing

Purified enzyme-cleaved RNA from 24 hpf zebrafish was adapter-ligated using Ion total RNA-Seq kit v2 (Life Technologies, USA) using manufacturer’s protocol. The phosphate groups from the 3′-ends of the alkaline hydrolysed enzyme-cleaved fragments were removed by Antarctic phosphatase treatment to generate 3’-OH ends for adapter ligation. The 5′ adapter ligated products were treated with 5 μl of 10× Antarctic phosphatase buffer (NEB), 2.5 μl of Superasin RNase inhibitor (Life Technologies, USA) and 2.5 μl of Antarctic phosphatase enzyme (NEB). The volume was made to 50 μl using nuclease free water (Ambion, USA). This was followed by adapter ligation at 3’OH ends generated after phosphatase treatment. The adapter ligated products were reverse transcribed to obtain cDNA and amplified by PCR to generate the sequencing library. At each step, purification was carried out using nucleic acid beads enrichment protocol compatible with standard sequencing techniques. The libraries were sequenced on Ion Proton platform (Life Technologies, CA, US) employing semiconductor based chemistry after quality check to generate single end reads.

### Data analysis

Sequencing was done for five technical replicates each for RNase V1 and S1 Nuclease probed samples (Additional file 1: Table S1).The raw single-end reads generated by Ion Proton sequencing were trimmed with BWA algorithm at a threshold of Q13 (*p-value* = 0.05) and length-sorted with a threshold of 25 nucleotides as implemented by SolexaQA version 2.2 [56]. The pre-processed reads were mapped back to the zebrafish transcriptome assembly downloaded from Ensembl (v79, Zv9) comprising of 56,754 transcripts (33,737 genes) using a two-stepped approach involving STAR aligner [57] and Bowtie2 [58] as prescribed by Life technologies (http://www.thermofisher.com/order/catalog/product/4476610?ICID=search-product&CID=fl-ion-proton-docs). First, the reads were mapped using STAR (with parameters --outReadsUnmapped Fastx --outSAMstrandField intronMotif). The unmapped reads obtained were then aligned locally using Bowtie2 (with parameters --local --no-unal -k 10). The bam files obtained from the above steps were merged using Samtools [59]. In order to select only uniquely mapped reads, those reads mapping more than once in the zebrafish reference genome (Zv9/danRer7) were removed. Aligned reads with erroneous read starts (5′ ends) were further removed to retain only high-confidence reads with perfectly aligned read starts.

### Calculation of load, position coverage and ratio scores

Load for each transcript was estimated by the total number of reads mapping to the transcript relative to the effective (mapped) length of the transcript. Load score determines the transcript abundance in the sample [36]. The transcripts with load ≥ 1 (atleast one read per base) were considered.

The position coverage for every transcript was also computed by summing the total number of positions with read starts obtained from both RNase V1 and S1 Nuclease data relative to the length of the transcript. The read starts define the enzymatic cleavage for the respective position and the pairing probability of the prior nucleotide as the RNase V1 and S1 Nuclease enzymes cleave at 3′ phosphodiester bond of the paired and unpaired position respectively. The transcripts that had load score of more than one and at least 85% positions covered with read starts were considered for further analysis.

Ratio score for every position in each of the RNase V1 (henceforth represented as V1 dataset) and S1 Nuclease (henceforth represented as S1 dataset) datasets were calculated by read start coverage for each nucleotide relative to the load of the transcript. Only those positions, which have more than one ratio score, were called as peaks. A peak could be present only in one of the dataset (V1 or S1) and confirmed that a position can be either paired or unpaired. If a position displayed a peak in both the (V1 and S1) datasets, it was termed as overlapping peak and corresponded to dynamic regions with multi-conformations, which were not able to acquire a stable structure. Any transcript with more than five such positions was termed as a multi-conformation transcript.

### Calculation of parallel analysis of RNA structure (PARS) scores

The number of reads initiating at every position in the transcriptome were calculated in both V1 and S1 datasets. Normalisation constants were calculated for both the datasets as K_v_ and K_s_ as per the following formula:

$$ {\mathbf{K}}_{\mathbf{v}}=\frac{\left(\mathbf{V}\mathbf{1}+\mathbf{S}\mathbf{1}\right)/\mathbf{2}}{\mathbf{V1}}\kern0.5em {\mathbf{K}}_{\mathbf{s}}=\frac{\left(\mathbf{V}\mathbf{1}+\mathbf{S}\mathbf{1}\right)/\mathbf{2}}{\mathbf{S1}} $$

*V1* and *S1* are total read starts for all positions covered by uniquely mapped reads in V1 and S1 datasets. Read counts for every position are further normalised by multiplying them by normalisation constants. This was done to eliminate the read disparity in the two datasets.

$$ \mathbf{V}{\mathbf{1}}_{\mathbf{i}}={\mathbf{K}}_{\mathbf{v}}\left(\mathbf{Raw}\;\mathbf{V}{\mathbf{1}}_{\mathbf{i}}\right)\kern0.5em \mathbf{S}{\mathbf{1}}_{\mathbf{i}}={\mathbf{K}}_{\mathbf{s}}\left(\mathbf{Raw}\;\mathbf{S}{\mathbf{1}}_{\mathbf{i}}\right) $$

PARS score for every position was calculated by the following formula.

$$ {\mathbf{S}\mathbf{core}}_{\mathbf{i}\hbox{-} \mathbf{1}}{=\mathbf{\log}}_{\mathbf{2}}\frac{\mathbf{V}{\mathbf{1}}_{\mathbf{i}}+\mathbf{5}}{\mathbf{S}{\mathbf{1}}_{\mathbf{i}}+\mathbf{5}} $$

Where *i* is any nucleotide position, PARS score for a position defines the pairing probability of the previous position.

### Enzymatic Footprinting

In vitro synthesised transcript of *ubiquitin C* UTR was generated using T7 Megascript kit according to manufacturer’s instructions (Life Technologies, USA). The RNA was checked for single RNA species using 12% denaturing polyacrylamide gel electrophoresis (PAGE). The composition of the gel was 40% 29:1 acrylamide:bisacrylamide, 8 M urea, 133.5 mM TBE. The gel mix is polymerized using 10% APS and 0.05% TEMED. The RNA products were visualized by UV shadowing and eluted from the gel using RNA elution buffer containing 300 mM Sodium acetate + 1 mM EDTA [60].

Gel purified RNA was radiolabelled using the Kinase max kit (Life Technologies, USA) as per the protocol provided by the manufacturer. Briefly, the RNA was dephosphorylated using Calf Intestinal phosphatase (CIP) and purified using Phosphatase Removal Reagent (PRR). The transcript was further incubated with T4 polynucleotide kinase and [γ-^32^P] ATP (BARC, India) for overnight at 37 °C [60]. The labelled RNA was further purified using NucAway columns (Life Technologies, USA).

5′-end-radiolabelled RNA (50,000 counts per lane) was added to 1 μg of unlabelled zebrafish RNA and was subjected to heating at 90 °C for 5 min and then allowed to cool to 28 °C in RNA structure buffer (Life Technologies) and 5 mM MgCl_2_ for overnight to facilitate structure formation. Folded RNA was subjected to digestion with RNase V1 (1:1600 U for 45 s) and S1 Nuclease (1:100 U for 1 min) at 28 °C respectively. RNA ladder for G residues was obtained by digesting the RNA with 1 U of RNase T1 (Fermentas) in the presence of 1 M LiCl and 100 mM MgCl_2_ for 2.5 min at 37 °C. Alkaline hydrolysis of RNA was performed at 90 °C in 0.5 M sodium bicarbonate buffer for 8 min. All reactions were stopped using equal volumes of gel loading buffer II (Life Technologies, USA) containing 95% formamide and 18 mM EDTA and snap-chilled on ice. Equal counts of digested products were separated on a 12% denaturing gel in 0.5× Tris-borate EDTA buffer and exposed to a phosphorimager screen. The gel images were scanned on a Typhoon scanner (GE Healthcare). Cleavage profiles were visualised using ImageQuant 5.2 software (GE Healthcare).

### Validation of PARS in zebrafish using *rpl35*

Several studies have highlighted that protein-coding genes are well conserved across species based on nucleotide sequence and function. The RNA secondary structures of such conserved genes are also known to be preserved. We tested the validity of PARS based pairing probability in zebrafish using a well-conserved protein-coding gene across human and zebrafish. Pairing probability of *rpl35* (*ribosomal protein large subunit 35*), a candidate gene encoding the protein component of 60S ribosome subunit was compared with its human homolog.

### Region-wise RNA structures across enriched gene ontology terms

Gene ontology enrichment analysis was performed for the transcripts showing position coverage of least 85% of the transcript length (209 genes) using WebGEStalt [61]. The enrichment analysis was performed for Molecular Functions gene ontology using default statistical test options and a significance level threshold of 0.05. In order to assess the extent of over-structuring or under-structuring of the RNA within the UTRs and CDS of the transcripts belonging to the enriched GO terms, we employed single sample Wilcoxon rank sum test (with mu = average PARS score for the respective regions - 5’-UTR, CDS and 3’-UTR). The resulting significant *p-values* were plotted as a heatmap for further inference.

### Periodicity pattern in coding regions of mRNAs

Periodicity across CDS regions was determined using Discrete Fourier Transform analysis [36]. PARS scores across first 100 positions in the CDS of 451 transcripts were averaged and were checked for periodicity.

### Codon-wise pairing probability

Average PARS scores of first 100 CDS positions were separated into 33 codons. Anova Test (not assuming equal variances) was used to affirm that the PARS score for every position in a codon differs from the rest of the two positions. If significant, pairwise comparisons of PARS scores using t-test with pooled Standard Deviation (SD) was performed.

### Structure probing of non-coding RNAs

Human *HOTAIR* [62] transcript with well-defined RNA secondary structure [19] was employed (approx. 1 picomole) as a positive control. *HOTAIR* was folded and probed at 37 °C with 10 μl of (1:1600 U) of RNase V1 for 45 s and 10 μl of (10,000 U) S1 Nuclease for 5 min. Similarly, RNA structures of two zebrafish candidate non-coding RNAs viz. *y-rna* and *tie-1as* were elucidated using PARS. Approximately, one picomole of the above mentioned in-vitro synthesised transcripts were pooled with zebrafish poly-A RNA to constitute 2 μg of the starting material. They were enzymatically probed by both RNase V1 and S1 nuclease as mentioned above and RNA libraries were prepared for sequencing. The data analysis was performed for the candidate ncRNAs using the pipeline described previously. The PARS scores were computed for each ncRNA using the method described in the above section. The oligo sequences used in the study are provided in Additional file 1: Table S2.

## Additional files

Additional file 1: Supplementary Tables and Figures. (PDF 454 kb)

Additional file 2: Load score and percentage coverage of 54,083 transcripts. (TXT 3635 kb)

Additional file 3: Multi-conformation position counts in transcripts with overlapping peaks. (TXT 712 kb)

Additional file 4: Number of read starts for every position covered in RNase V1 sample. (TXT 32051 kb)

Additional file 5: Number of read starts for every position covered in S1 nuclease sample. (TXT 45980 kb)

Additional file 6: Total number of read starts for every position in both RNase V1 and S1 nuclease sample. (TXT 64960 kb)

Additional file 7: Positions with ratio score more than one in V1 dataset. (TXT 15410 kb)

Additional file 8: Positions with ratio score more than one in S1 dataset. (TXT 15865 kb)

Additional file 9: PARS scores of 54,083 transcripts and non-coding RNAs. (TXT 35418 kb)

## Abbreviations

CDS: Coding DNA Sequence
DMS: Dimethyl sulfate
GO: Gene Ontology
h: Hours
min: Minutes
PARS: Parallel Analysis of RNA Structure
s: Seconds
SD: Standard Deviation
SHAPE: Selective 2’-Hydroxyl Acylation Analyzed by Primer Extension
SNV: Single Nucleotide Variation
UTR: Untranslated Region
