# Supplementary material for: RNA secondary structure profiling in zebrafish reveals unique regulatory features
Source: BMC Genomics. 2018 Feb 15;19:147. doi: 10.1186/s12864-018-4497-0 (PMC5815192; doi:10.1186/s12864-018-4497-0)
Supplement: Supplementary file 1 — Supplementary Tables and Figures. (PDF 454 kb) [file 12864_2018_4497_MOESM1_ESM.pdf]

SUPPLEMENTARY TABLES AND FIGURES

Figure S1: Gel picture showing RNase V1 and S1 nuclease cleaved RNA library of 24hpf ASWT zebrafish. Libraries of size 150-500 bp were obtained upon PARS.

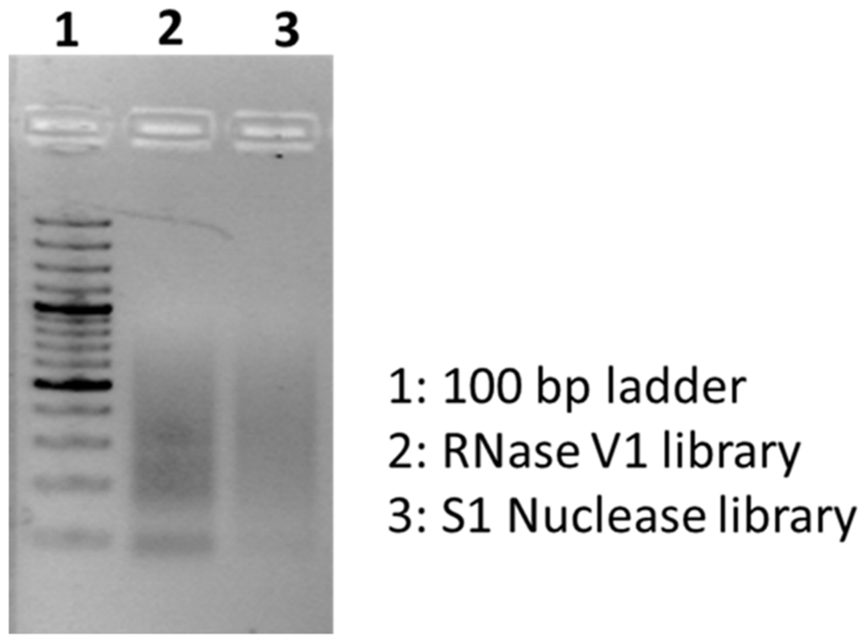

Table S1. RNA-seq data production and alignment results for 24hpf zebrafish poly (A) reads.

	Raw reads	Trimmed reads	Mapped reads to genome	Mapped reads to transcriptome	Unique reads
V1_1	55,155,258	49,909,300	43,148,377 (86.45%)	18,591,806 (37.25%)	32,312,362 (64.74%)
V1_2	25,178,053	13,967,707	11,076,489 (79.3%)	3,331,732 (23.85%)	8,252,309 (59.08%)
V1_3	38,155,820	30,222,770	26,728,488 (88.44%)	11,973,454 (39.62%)	20,178,004 (66.76%)
V1_4	44,573,054	34,968,487	30,871,536 (88.28%)	13,312,744 (38.07%)	22,882,036 (65.43%)
V1_5	41,897,201	32,072,740	28,337,847 (88.35%)	12,522,739 (39.04%)	21,228,418 (66.18%)
V1_Total	204,959,386	161,141,004	140,162,737 (87%)	59,732,475 (37.06%)	104,853,129 (65.07%)
S1_1	46,580,204	36,883,629	34,927,290 (94.7%)	23,541,466 (63.82%)	28,578,825 (77.48%)
S1_2	48,723,018	45,017,999	43,038,016 (95.60%)	30,464,719(67.67%)	36,987,573 (82.16%)
S1_3	27,629,374	23,092,834	21,229,900 (91.93%)	12,477,230 (54.03%)	17,005,650 (73.64%)
S1_4	39,573,890	33,136,122	30,678,033 (92.58%)	18,169,500 (54.83%)	24,423,436 (73.71%)
S1_5	50,540,637	42,640,345	39,802,137 (93.34%)	24,381,843 (57.18%)	32,175,247 (75.45%)
S1_Total	213,047,123	180,770,929	169,675,376 (93.86%)	109,034,758 (60.32%)	139,170,731 (76.9%)

Table S2: List of oligo sequences used in the study

S.No	Gene Name	Forward primer	Reverse primer
1	ubc	TAATACGACTCACTATAGGGTCTGCTTATGATTAATCATTAATATATACAAGTCTTGAATTATCAGACCA	TCTGGAATGCAAGAACTTTATTGAATGATTAAGAGGAATAAAGGAAACTGGAATTGGTCTGATAATTCAAGACTT
2	HOTAIR	ACTCGCCTGTGCTCTGGAGCTT	TACACAAAGTGCATACCTACCC
3	y-rna	TAATACGACTCACTATAGGGGGCTGGTCCGAAGGCGGTGGGTTAGTCACAATTGATTGCTACAGTCAGTTACAGAA	AAAAGGCTGGTCAAGTTCAGCGGTGGGAGAGGGGAGAGAAGAAACAAGGAGTTCTGTAACTGACTGTAGCAATCAA
4	tie1-as	GGCCACCAAGGACAGATTCA	TCTTTGGCATTGGTGTGGAC

Supplementary Methods

RNA Sequencing parameters

All the libraries were barcoded using standard Ion Xpress RNAseq barcode 0-16 kit prior to sequencing. We have used Ion Proton standard Ion PI 200v3 sequencing chemistry and Ion PI chip v2 for generating the data by following manufacturer-supplied protocol. The read length for the sequencing was set as 200 with a 500 flow cycle. The flow order and quality score calculation matrices were default as per the Torrent Suite v.4.0.1 run quality. The default flow order and library keys were TACGTACGTCTGAGCATCGATCGATGTACAGC and TCAG respectively.

5 µL of Ion PI control ion sphere particles (control ISPs) were added to the purified sequencing ISPs (ie, the control ISPs ratios were maintained ~2% to the positive sequencing ISPs) for assessing the overall sequencing quality. The successful run was defined with a maximum polyclonality of ≤ 30%, maximum control ISPs of ≤ 2.5% and with a minimum of 90% (percentage 50AQ17) control ISP read's first 50 bp sequenced with an error rate of 1 in 50, Phred like score 17 or higher. The remaining quality trimming issues with actual sequencing sample reads were assumed to be associated with the samples. The read trimming during sequencing was set to be false during the sequencing to preserve the first base of the sequencing reads. All the reads binned according to the barcode by the Torrent Suite using default parameters were first trimmed for the Ion RNA barcode.
